# Superman vs. Nick O’Teen: anti-smoking campaigns and children in 1980s Britain

**DOI:** 10.1057/s41599-019-0326-6

**Published:** 2019-10-01

**Authors:** Alex Mold, Hannah Elizabeth

**Affiliations:** 1 London School of Hygiene and Tropical Medicine, London WC1E 7HT, UK

## Abstract

In December 1980, the Health Education Council launched a campaign designed to discourage children from taking up smoking. Advertisements on TV and in comics and magazines featured a battle between Superman and the evil Nick O’Teen as he attempted to recruit children to his army of smokers. Children were also encouraged to join Superman in his fight by signing a pledge not to smoke, in return for which they received a poster and badges featuring the superhero. This article examines the design, production, delivery and reception of the Superman vs. Nick O’Teen campaign in order to probe the multi-faceted nature of the making of healthy publics in 1980s Britain. Children constituted a particularly problematic public. On the one hand, they were thought to be vulnerable and easily led towards unhealthy lifestyle choices. But on the other, children were also recognised as agents who might convince adults, as well as their peers not to smoke. This ambivalent conceptualisation of the child as a potential victim of malign influences, or potential rational agent and force for good, is typical of the 1980s, a time when the meanings of the child as consumer, agent, and citizen were undergoing increased ideological debate. This campaign also took place as ideas about health education, its place within public health policy and practice, and its relationship with the public, were in flux. The battle between Superman and Nick O’Teen was thus not just about smoking, but about particular ways of seeing and interacting with healthy (and unhealthy) publics.

## Introduction

On Boxing Day in 1980, an anti-smoking advertisement paid for by the Health Education Council (HEC) and designed by the advertising agency Saatchi and Saatchi, aired on British televisions for the first time. The 30-s clip showed ‘Nick O’Teen’ attempting to encourage a group of children to start smoking, only to be thwarted at the last minute by Superman, who swoops in and throws Nick O’Teen and his cigarettes into the distance. Superman’s X-ray vision, he tells viewers, allows him to see inside people’s bodies which is why he ‘Never says yes to a cigarette’ (Superman vs. Nick O’Teen, 1980). The TV advertisement was part of a campaign run by the HEC from 1980 until 1982, costing in excess of £3.5 million. A wide range of visual sources, including posters, comic books and badges were put to use to encourage children aged 7–11-years-old to join Superman in his fight against Nick O’Teen. The HEC judged the campaign to be a victory, pointing to the fact that 800,000 children requested an anti-smoking pack and 92% of children surveyed had retained the poster featuring the superhero (Jacob, 1985). Yet, if the campaign’s aim was to discourage children from smoking, its success or otherwise was much more difficult to judge. Smoking rates amongst children and young people remained stubbornly consistent at around 13–10% throughout the 1980s and 1990s, and only started to fall markedly in the 2010s (ASH, 2018). Like many other health education campaigns then and since, it is almost impossible to assess whether or not Superman was able to defeat Nick O’Teen in the long-run.

This does not mean, however, that these efforts were without interest or value. Indeed, we argue that the Nick O’Teen campaign can tell us much about the multi-faceted nature of the making of healthy publics in 1980s Britain. The place of the public in post-war public health, and in health education and health promotion in particular, was a hotly contested issue. As Mold and colleagues suggest, there was not one ‘public’ but many. There were collective understandings of publics as populations or citizens, but publics were often fragmented into traditional groupings such as socio-economic status, gender and ethnicity. Publics were also able to ‘speak back’ to public health authorities through active and more passive forms of resistance. Ideas about the meaning of publicness also changed over time, as the boundary between the public and private in health fluctuated (Mold et al., 2019). ‘The public’ was not a population waiting to be discovered or a target for intervention, but, as Hinchcliffe and colleagues suggest, can be better thought of as ‘healthy publics’ consisting of ‘dynamic collectives of people, ideas and environments that enable health and well-being’ (Hinchliffe et al., 2018, p. 2).

This article develops and extends the notion of healthy publics as multiple, active and dynamic in two ways. Firstly, it considers how children constituted a particularly problematic public. On the one hand, they were thought to be vulnerable and easily led towards unhealthy lifestyle choices. But on the other, children were also recognised as agents who might convince adults, as well as their peers not to smoke. This ambivalent conceptualisation of the child as both a potential victim of malign influences, and a rational agent and force for good, is typical of the 1980s, a time when the meanings of the child as consumer, agent, and citizen were undergoing increased ideological debate. This campaign also took place as ideas about health education, its place within public health policy and practice, and its relationship with the public, were in flux. The battle between Superman and Nick O’Teen was thus not just about smoking, but about particular ways of seeing and interacting with healthy (and unhealthy) publics. The second contribution this article makes is to demonstrate that even problematic publics like children had agency and were not simply passive recipients of public health interventions. This was reflected in the HEC’s willingness to involve children in the design of the campaign and the propagation of its message. This displayed a capacity to see that the public needed to be part of the way such campaigns operated. Children, as a particular public with specific vulnerabilities and capacities, were at the frontline of a process within health education that had begun to take seriously the agency of the publics it was attempting to reach.

This article begins by setting the Nick O’Teen campaign in context, through a consideration of health education efforts around smoking from the 1960s onwards and those targeted at children in particular. We also examine the broader health education programmes directed at children to draw out some of the wider themes at work in the conceptualisation of children as a public. We then move on to analyse the Nick O’Teen campaign, focussing on its aims and objectives and on the design and content of the materials produced. The posters, comic strip and other material reveals a view of children as a problematic public, but one with a degree of agency. This leads us to reflect on the nature of health education and its conceptualisation of the public or publics, during the 1980s. We suggest that this was a moment of transition, as ideas about ‘the public’ and ‘healthy publics’ began to shift.

## Smoking, health education and children

The notion that smoking was potentially dangerous to health and to morality was not confined to the 1980s. In the early twentieth century, juvenile smoking prompted particular concern, leading to the introduction of restrictions on the sale of tobacco to children through the Children’s Act in 1908 (Hilton, 1995). Worries about the danger to health were wrapped up with a broader set of fears about delinquency, hooliganism and physical deterioration (Welshman, 1996). However, it was the identification of a link between smoking and lung cancer in the 1950s that prompted the first consistent attempts to discourage adults and children from smoking. The early response from British health educators was fairly muted, and as Berridge and Loughlin point out, mostly confined to the local level (Berridge and Loughlin, 2005). During the 1960s, the volume of anti-smoking health education material increased significantly. This was for two reasons. Firstly, the medical profession and health policymakers now accepted the link between smoking and lung cancer, as well as the need to take action. The publication of the Royal College of Physician’s report, *Smoking and Health* in 1962 was a key moment. The report demonstrated a new willingness on the part of medicine to speak to the public and a recognition that the mass media was a valuable tool for improving public health. At the same time, the report put forward a view of the public as a collection of individuals who needed to change their behaviour (in this case give up smoking) to benefit public health (Berridge, 2007a). *Smoking and Health* also asserted that a particular group in need of education were children, in order to prevent them from taking up smoking. The second factor behind the growth of anti-smoking material was a wider push towards making greater use of health education to encourage behaviour change. The 1964 Cohen report asserted that health education should do more than provide information: it should ‘seek to influence people to act on the advice and information given’ (Central Health Services Council and Scottish Health Services Council, 1964, p. 9). The report also drew attention to the need for more health education in schools, and on smoking in particular.

Beginning in the early 1960s, there were a number of efforts to reach children and young people with anti-smoking messages. In 1962–1963, a van sponsored by the Central Council for Health Education drove around the country distributing anti-smoking material (Berridge, 2007b, pp. 72–73). Poster campaigns frequently featured young people and in 1967 the Central Office for Information released a film targeted at teenagers. ‘Dying for a smoke’ was made by the animators Halas and Batchelor, and featured ‘Old Nick O’Teen’, a motorbike-riding devil figure who attempted to recruit ‘Sam Sucker’ and his friends to a chain-gang of smokers (Halas and Batchelor, 1967). By the 1970s, there was a further upswing in the volume of anti-smoking material and also a change in its design and approach. The Cohen report had resulted in the establishment of national bodies in charge of health education, the Health Education Council (HEC) in England and Wales, and the Scottish Health Education Group in Scotland. After a brief foray into designing their own material, the HEC decided to make use of professional advertising agencies and especially the emerging firm, Saatchi and Saatchi. The agency came up with a number of eye-catching campaigns, such as that featuring a naked pregnant woman smoking. Children featured obliquely in this campaign, as babies that were put at risk by maternal smoking, the unborn child deployed as a metaphor for a threatened future rather than targeted as a possible public receptive to health education. While other campaigns targeted young people directly, such as a poster featuring the rock star Mark Bolan, campaigns prior to the 1980s tended to concentrate on either the threat of adult smoking to child health, or on teenagers who were envisaged as being capable of making their own decisions around smoking.

The lack of direct interventions aimed at young children and smoking before 1980, despite the potential health threat it represented, paralleled a more general pattern in health education featuring children across the twentieth century. Children often appeared in public health campaigns as metaphors for the future or as symptoms of adult failure. Children, therefore, served more as reminders for adults to behave as good healthy citizens, than as the primary audience or beneficiary. Frequently, even those health campaigns designed to improve or protect the health of children targeted children’s guardians rather than children themselves (Elizabeth et al., n.d.). These efforts ranged from preventative health interventions like campaigns around vaccination, sexual health, or dental care, to more nebulous efforts to prevent moral or physical injury by advertising the dangers of roads, pre-marital sex, kitchens, strangers, quarries, and rubbish dumps, to name but a few. For example, the 1971 cartoon film *Children and Disused Fridges*, warned adults to dispose of fridges responsibly in order to prevent children playing inside abandoned fridges from suffocating (Children and Disused Fridges, 1971). Other films, such as the ‘Charley Says’ series, warned children of threats to their wellbeing more directly, rather than asking adults to protect them (Crane, 2018). However, unlike health campaigns which targeted adults as individuals capable of making unhealthy choices, rendering the adults themselves a threat to the nation’s health, the threats featured in these campaigns were positioned as external to the children. Such campaigns imagined children as having enough agency to avoid immediate obstacles to their wellbeing, but rarely assumed pre-teens had sufficient capacity to be threats, or comprehend threats, to the future. For example, as Joe Moran demonstrated in his analysis of 1960s and 1970s road safety campaigns, those directed at children were dependent on a view of the child as capable of learning and applying certain principles to avoid threats when crossing the road (Moran, 2006).

The variable nature of campaigns targeted at protecting or improving the health of children was not limited to their subject matter but also their quality. Indeed, campaigns which had children among their target audiences often included children’s health education as an addendum to adult health education. Child-specific campaigns were only deployed when reaching children through their guardians was deemed inadequate or unsuitable (Elizabeth, 2016). While some campaigns did dual service, targeting adults and children as publics together, these tended to focus on older children where comprehension and agency, and so ability to both threaten and guard health, was likely to be seen as akin to that of an adult. While sexual health education offers some examples of carefully designed curricula aimed at those below the age of majority, the controversy which swirled about sex education during the 1980s makes it an ill-fitting comparison for anti-smoking campaigns. Moreover, sexual health education aimed at the young was, until the late 1980s, produced almost entirely out of house by sub-contracted charities rather than government bodies, kept at arms-length by the government to avoid being embroiled in the controversies it provoked (Berridge, 1996, p. 7; Meredith, 1989, p. 82). Nick O’Teen, as a carefully constructed and multifaceted education campaign aimed entirely at children, and intended to mobilise the young to reach the old, seems to be a marked departure from the ad-hoc and improvised health education which was normally produced for those below the age of majority in the twentieth century.

A desire to reach children with anti-smoking messages targeted at them specifically was prompted by research that appeared to indicate that smokers took up the habit at an early age. In 1977, the Royal College of Physicians published a report, *Smoking or Health*, that asserted that some children started smoking as young as age 5, and one in three regular smokers had taken up smoking before the age of 9 (Royal College of Physicians, 1977). A junior minister at the Department of Health and Social Security, Sir George Young, was especially concerned about these statistics so he secured £500,000 for the HEC to mount an anti-smoking campaign targeted at children, and this formed the basis for the Nick O’Teen campaign (Health Education Council, 1980a; House of Commons, 1980). The turn to health education rather than any other method of preventing children from taking up smoking was in line with the policy response to children’s smoking from the 1960s through to the 1990s. Although restrictions on the sale of tobacco to children had been in place since the early twentieth century, calls to tighten up legislation were controversial, and education and voluntary codes of practice on advertising prevailed (Berridge, 2007a). Legislation was introduced in 1986 to prevent the sale of any tobacco product to children; previously the restrictions only applied to smoking tobacco. During the 1990s, partial bans on advertising in publications and places likely to be seen by children were gradually introduced. Public health policy makers and practitioners were concerned with the exposure of foetuses and children to tobacco smoke, resulting in efforts designed to reach pregnant women and parents and persuade them to stop (Berridge, 2007b). Health education, of both adults and children continued (and continue) to play a central role in the response to tobacco smoking. The Nick O’Teen campaign was thus of a piece with wider anti-tobacco policy and health education efforts, but also took this in new directions.

## The Nick O’Teen campaign: aims and design

The Nick O’Teen campaign built on existing work carried out by the HEC. According to Ian Sutherland, Director of Education and Training at the HEC from 1970–1985, the Council were already developing a children’s anti-smoking campaign featuring Superman (Sutherland, 1987, pp. 90–91). In 1979, the HEC piloted a poster and full-page advertisement in children’s comics depicting Superman and the message ‘With my amazing X-ray vision I can see the harm cigarettes do inside people’s bodies. That’s why I don’t smoke.’ Micheál Jacob, a press officer at the HEC, reported that the council received over 70,000 requests for the poster, and the government money meant that the campaign could be expanded considerably (Jacob, 1985). The new Nick O’Teen campaign was intended to target children aged 7–11 years. A creative brief written by the HEC for Saatchi and Saatchi asserted that ‘We need advertising which states in simple terms to which children will relate that smoking is not a necessary adjunct to adult, cool or heroic behaviour. The health dangers inherent in the habit should be a secondary part of the message.’ (Saatchi and Saatchi, 1979) This statement reveals much about the way the HEC viewed children, their attitudes towards smoking and how they could be persuaded not to take up the habit. The underlying assumption behind the counter-narrative about smoking—that it was ‘not a necessary adjunct to adult, cool or heroic behaviour’—suggests children already held this as a belief, which they then needed to be disabused of. Health dangers were addressed as a secondary message because they were presumed to be more boring to children and therefore less persuasive. The HEC were aware that children could not simply be educated about the dangers of smoking for prevention to be successful, but that their prior knowledge and existing emotions related to cigarettes, their peers, and education, needed to be addressed, mobilised, and changed if necessary. This came through strongly in the aims of the campaign, which were summarised by David St George, a research officer at the HEC. He stated that ‘The aims of the Superman campaign are to resonate with and strengthen anti-smoking attitudes which already exist in the target group, and to help them subsequently resist peer-pressure by providing them with an imaginary role-model to which they can relate’ (Health Education Council, 1980b). But the campaign aimed to go beyond building on existing attitudes: it wanted to encourage active participation by children in propagating and strengthening the anti-smoking message. Freddie Lawrence, Chief Information Officer at the HEC said that the campaign was intended to: ‘a) reinforce existing attitudes already favourably disposed to anti-smoking; b) “enlist” their active participation in a frank battle between “good” and “bad” rather than merely give information and c) use the opportunity to communicate fairly sophisticated health messages to an audience whose future smoking behaviour will be determined to a great extent by their attitudes and knowledge now’ (Health Education Council, 1980c). The HEC viewed children as agents with the capacity to act independently, but they were also presumed to be particularly affected by their emotions. Fighting the “glossy” smoking image’, Lawrence argued, would take more than the potentially ‘dull and authoritarian’ ‘[h]ealth education messages’ because as he explained: ‘It is easier to sell the delights of chocolate bars, and persuade children to go out and buy them, than it is to sell them the concept that “smoking is bad” …However, I believe HEC has demonstrated that dull topics like these can be presented in such a way as to excite children’s interest and focus their attention on our messages’ (Health Education Council, 1980c). Lawrence here framed children as emotional creatures—delighted by chocolate, bored by ‘dull topics’, and requiring excitement if their ‘interest and focus’ was to be gained and kept. Underlying this assessment of the HEC’s young audience though, is the assumption that just as ‘glossy’ smoking adverts could garner the attention of children, so too could HEC health messages.

The HEC’s desire to deliver a ‘glossy’ campaign can be seen in its design and especially the decision to feature Superman. The use of comic book heroes to deliver propaganda messages to children was not a new idea—the Hulk had been deployed in the past by the HEC to encourage dental hygiene (Health Education Council, No date) —but considerable thought went into the HEC’s choice of Superman for this particular campaign. A report commissioned by the HEC to evaluate the pilot project and specifically the use of Superman found that he ‘was considered to be the most appropriate character of all the super heroes to represent good against bad and advise children not to smoke.’ Research ‘demonstrated that that he is an acceptable and exciting figure to small children’ (Health Education Council, 1980c). The decision to use Superman was not just based on evidence, but on a set of assumptions about the character and how young children would respond to this. An anonymous and undated note on the campaign suggested that Superman was ‘a good guy without being soft. He’s timeless, incorruptible and admired by kids and by using an existing character to which children can relate we get over the problem of handing down authoritarian messages from adults’ (Health Education Council, No date). By predicting their audience of children would be unreceptive to ‘authoritarian messages’ and a ‘soft’ persona, but would be enticed by a ‘timeless, incorruptible’ figure, the HEC was making assumptions about what children wanted. They anticipated a certain degree of anti-authoritarian sentiment and existing favourable attitudes to Superman, but also, despite the brief stating that ‘equal weighting should be given to boys and girls’, there was a rejection of ‘good guy’ obedience which could be mistaken for softness, a traditionally feminine characteristic. Concerns over how children would respond to Superman’s ‘good guy’ persona demonstrate some awareness of the growing popularity, since the late 1960s, of dystopian science fiction and anti-heroes in British and American children’s media (Braithwaite, 2010, p. 6). From Superman’s first appearance in June 1938, the character was criticised for being both too good, and so a little dull, and for being a thinly veiled vessel for saccharine American propaganda (Saunders, 2011, pp. 17–27; Valencia-García, 2012, pp. 45–47). These concerns could largely be dismissed as representing more of a trend in older adolescent consumption patterns, rather than being reflective of the tastes of the 7–11-year-olds targeted by the Nick O’Teen campaign. Nonetheless the caped hero remained a problematic choice, carrying myriad connotations over which the HEC had next to no control.

While children liked Superman, as a recognisable American hero, and one famed in part for his fighting prowess, he personified many adults’ anxieties about declining standards in British children’s media circulating at this time. Many private schools rejected the Superman packs because they did not like the comic book format (Jacob, 1985). Such rejections were symptomatic of wider anxieties in the 1980s about the media consumption habits of children, with concerns frequently expressed in the adult press about the content and style of children’s television, with worries that it was being ‘dumbed down’, or becoming ‘too American’ and ‘too violent’ (Buckingham, 1999, p. 7). Worries such as these hark back to admonitions about the ‘violence and triviality’ of broadcasts voiced in the 1960s during the discussions which were framed by the 1962 Pilkington Report on the future of broadcasting (Cabinet Office, 1962; House of Lords, 1962). They also echoed even earlier American moral panics about comic books themselves (Lent, 1999, pp. 9–17). Such anxieties were articulated with renewed vigor in the 1980s.

## Superman vs. Nick O’Teen–the materials and their meaning

Although the Nick O’Teen vs. Superman campaign appeared at a time when concerns about children’s media were particularly fraught, the HEC sought to utilise some of these very same modes to reach their target audience. The campaign was a multi-pronged effort that made use of a range of different media and materials. Alongside the 30-s TV cartoon commercial which aired over Christmas 1980, there were full-page advertisements that featured in range of children’s comics and magazines that ran from 11 January 1981 for 10 weeks. The HEC had already paid DC Comics a licence fee to use Superman’s image for their earlier campaign, so most of the new budget was spent on the TV advertisement, which cost £417,500 to produce (Health Education Council, 1979). The press adverts cost £32,5000 to produce. These appeared in magazines that targeted boys, such as *Marvel* and *Roy of the Rovers*; girls, such as *Jinty* and *Oh Boy*; and both boys and girls, such as *Look In* and *Whizzer and Chips* (Saatchi and Saatchi, 1980).

The magazine-based advertisements included an invitation for children to join Superman in his fight against Nick O’Teen. Children were asked to fill in a coupon with their name and address, and in return they would receive a pack containing: a poster; an eight-page comic book; a badge; an individually-numbered certificate stating that they had joined Superman in his fight against Nick O’Teen; and the chance to enter a poster-making competition where successful entrants could win prises, including a Raleigh bicycle. The packs were also sent to 21,000 primary schools. The invitation to send off for a Superman pack, the encouragement to enter a poster competition with prizes and to sign a certificate indicating they had joined the ‘fight against Nick O’Teen’ was illustrative of a participative approach to involving children in the campaign itself. In return for these actions, children received something tangible—a poster, certificate, comic book, a prize—rather than merely gaining a sense of accomplishment at having participated in some form of healthy citizenship. These actions did more than reinforce the anti-smoking message delivered by the overall campaign, or provide evidence of participation in a collective non-action (the refusal of smoking), they acted to recruit children into what was later described as an ‘anti-smoking lobby’ (Health Education Council, 1982). This created a sense that children, and their peers, had the power (and so a duty) to prevent smoking. This recruitment, into Superman’s anti-smoking army, hints at a perception of children as a potentially powerful force. Placed in comparison with earlier health campaigns featuring children, Nick O’Teen, in imagining children as guardians of their own health in the present and future, and as potential citizen lobbyists on behalf of the anti-smoking campaign, presents a marked departure from earlier more passive imaginings of children with limited agency under constant external threat. However, discussions within the HEC about the campaign, its planning, and its impacts, demonstrate a sustained ambivalence towards children’s agency and potential gullibility.

**Fig. 1:**
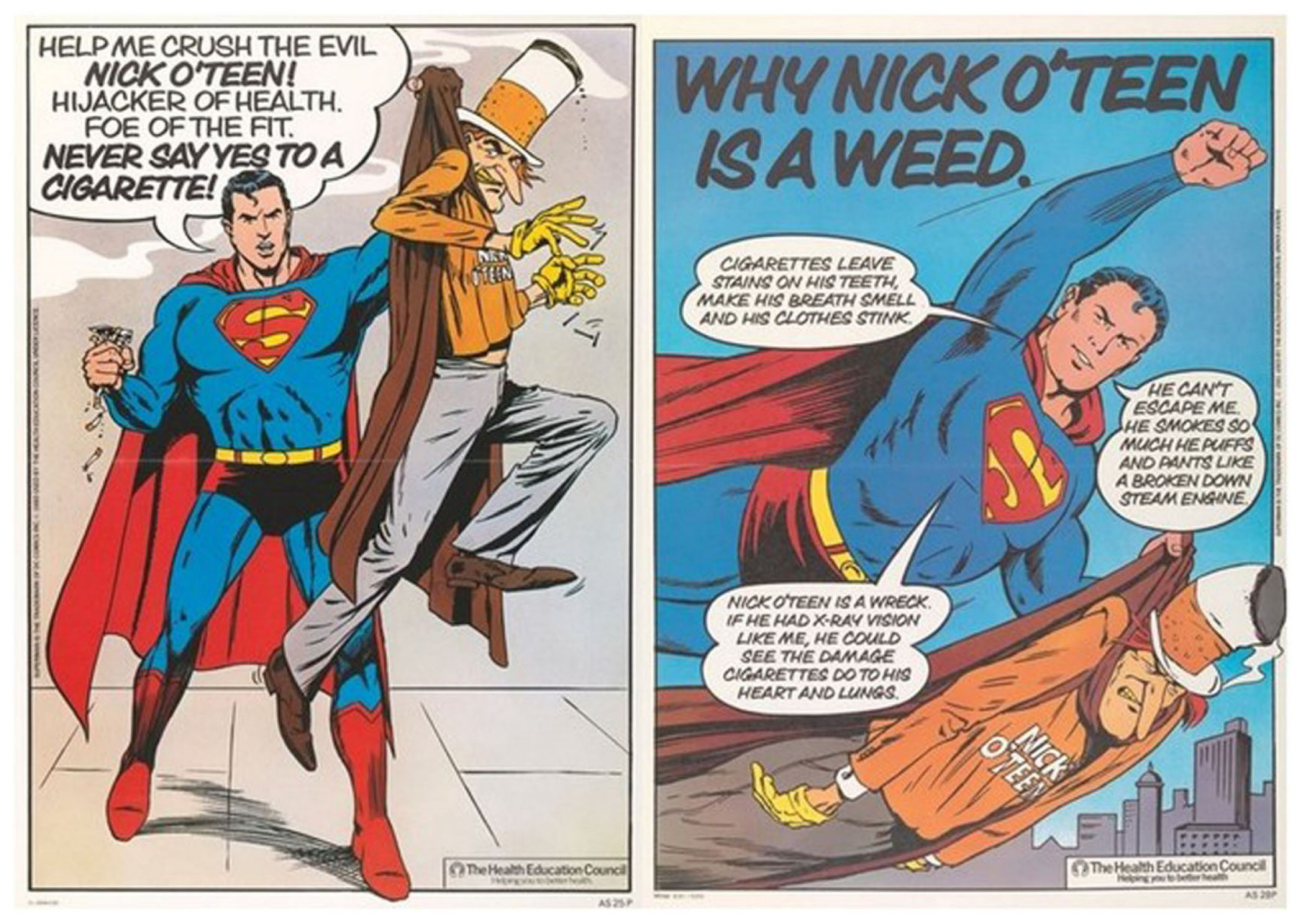
Nick O’Teen Posters. Saatchi and Saatchi for the Health Education Council, 1980a, 1980b, 1980c. This figure is covered by the Creative Commons Attribution 4.0 International License. Reproduced with permission of Crown; copyright © Crown, all rights reserved. This information is licensed under the Open Government Licence v3.0. To view this licence, visit http://www.nationalarchives.gov.uk/doc/open-government-licence/. Image courtesy of the Wellcome Collection

The HEC’s attitudes to children as a public were on display in the campaign itself. Children’s media, especially didactic products such as Nick O’Teen, reveal what adults envisioned childhood was or could be because they required an adult to first construct an imagined child as an audience to be aided by the text (Lesnik-Oberstein, 1999). Space precludes examining the campaign’s materials in their entirety, but an analysis of some of the images produced tells us much about how children were viewed. While Superman was the creation of DC Comics, Nick O’Teen was the product of an HEC consultation with focus groups of children. These children dubbed the HEC’s first attempt at Nick O’Teen’s image ‘insufficiently evil’, resulting in changes (Jacob, 1985, p. 16). The HEC was willing to take the desires and expertise of children seriously, suggesting a view of children as active participants and not simply a group to be instructed. The resultant Nick O’Teen is curious bricolage of child-catcher tropes; the cigarette-top stove-pipe hat, dingy cloak and yellow gloves lending him a distinctly Victorian villain aesthetic, hinting at parallels between Nick O’Teen and depictions of Fagin from Charles Dickens’ *Oliver Twist* (Fig. 1). In reaching for a salient fight between good and evil, the HEC and the children they consulted seem to have generated an image of stranger-danger with anti-Semitic undertones encapsulated by Dickens’ most famous Jewish villain. This characterisation of evil, as a persuasive and untrustworthy adult stranger, was not mere happenstance. While such threatening characters have appeared in children’s media for centuries, fears around ‘stranger-danger’ began to rise in the 1970s, with the grizzly details of child murders and exploitation making the press with increased fever as the twentieth century progressed (Crane, 2018). These factual rather than fictive narratives, while primarily circulating in an adult press perpetuating a characterisation of childhood as time of increasing vulnerability, were also disseminated to children through education media targeted at negating a perceived increased threat of ‘stranger danger’ (Erulkar, 1971). This characterisation of Nick O’Teen as a dangerous stranger, grooming children to smoke (while aesthetically implying worse) did more than convey evil, it also provided structure and an emotional pallet for the Superman vs. Nick O’Teen narratives across the entire campaign. This allowed for the redeployment of familiar external threat narratives which positioned children as agents able to vanquish a dangerous foe, but avoided any more complex suggestions that children, as would-be smokers, were themselves potentially a threat to health.

**Fig. 2:**
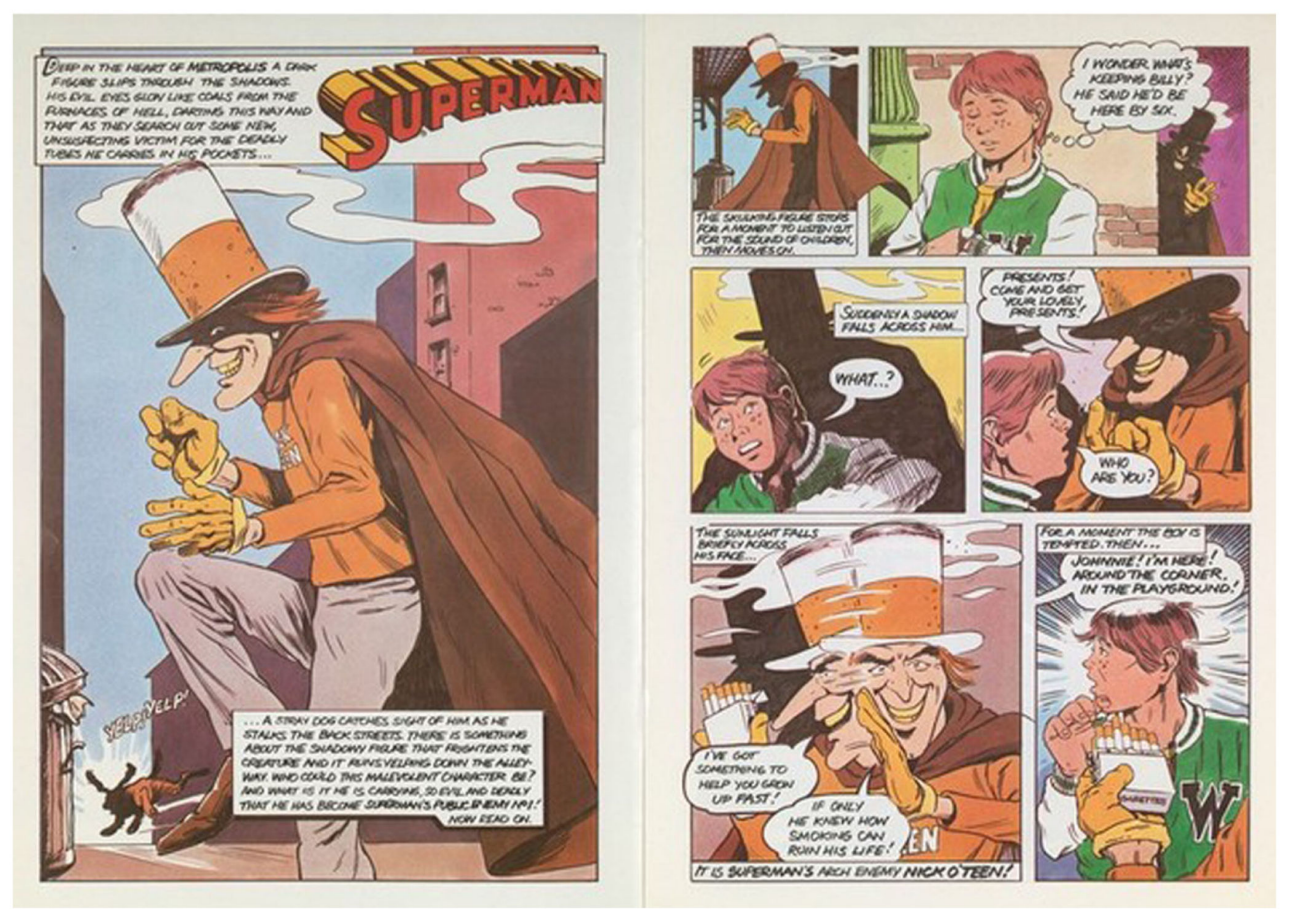
Pages 2 and 3 of the Superman vs. Nick O’Teen comic. Saatchi and Saatchi for the Health Education Council, 1980a, 1980b, 1980c. This figure is covered by the Creative Commons Attribution 4.0 International License. Reproduced with permission of Crown; copyright © Crown, all rights reserved. This information is licensed under the Open Government Licence v3.0. To view this licence, visit http://www.nationalarchives.gov.uk/doc/open-government-licence/. Image courtesy of the Wellcome Collection

**Fig. 3:**
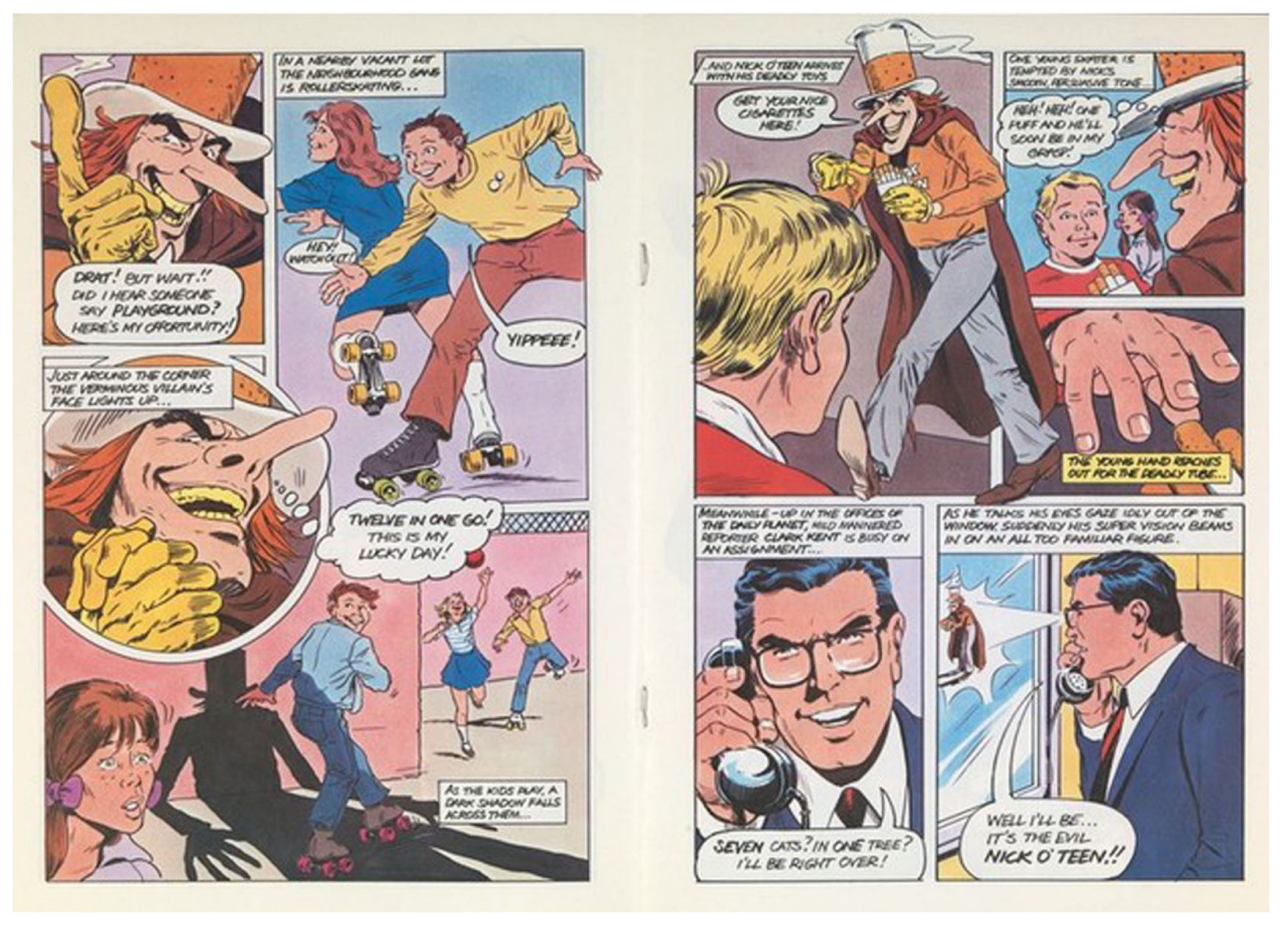
Pages 4 and 5 of the Superman vs. Nick O’Teen comic. Saatchi and Saatchi for the Health Education Council, 1980a, 1980b, 1980c. This figure is covered by the Creative Commons Attribution 4.0 International License. Reproduced with permission of Crown; copyright © Crown, all rights reserved. This information is licensed under the Open Government Licence v3.0. To view this licence, visit http://www.nationalarchives.gov.uk/doc/open-government-licence/. Image courtesy of the Wellcome Collection

The complexities surrounding the agency of children as a public can be observed in greater depth in the Superman vs. Nick O’Teen comic book. Nick O’Teen first appears in the Superman vs. Nick O’Teen comic book skulking about a back-alley of the ‘metropolis’, ‘his evil eyes …Darting this way and that as they search for their next unsuspecting victim for the deadly tubes [cigarettes] he carries in his pockets…’ Nick O’Teen’s evil credentials are established by his shifty behaviour, and the fear he inspires in a stray dog (Fig. 2). Nick O’Teen’s ‘skulking figure’ stops to ‘listen out for the sound of children’ before he comes across a lone boy, Johnnie. He implores Johnnie to ‘come and get your lovely presents’. Nick O’Teen then claims ‘I’ve got something to help you grow up fast!’ before whispering beneath a yellow gloved hand ‘If only he knew how smoking can ruin his life!’ Johnnie is depicted reaching for a cigarette, persuaded by the cajoling of Nick O’Teen and the enticement of accelerated adulthood he promises, only to be saved by the calls of his friend at the last minute. Thwarted, Nick O’Teen is seen approaching a playground, where ‘one young skater is almost tempted by Nick O’Teen’s smooth persuasive tone’, his ‘young hand reaches out for the deadly tube’, while Nick O’Teen thinks ‘heh heh, one puff and he’ll soon be in my grasp!’ (Fig. 3). This pairing of children’s desire to appear mature with a future ruined by smoking continues throughout the comic, reflecting the HEC’s intention that the campaign should make clear ‘smoking is not a necessary adjunct to adult, cool or heroic behaviour.’ The secondary preventative health message, that smoking damages health and leads to addiction, is emphasised by Nick O’Teen’s apparent infirmity–his lanky frame and stained teeth lying in sharp contrast to the superhuman healthy figure of Superman and his pearly-white teeth.

The emphasis on growing up also casts the two adult characters in the roles of divergent futures: one offering access to the role of a powerfully healthy non-smoking citizen, participating in society and defending justice; the other a sick, itinerant, addict, outsider, so rejected by society he must adopt disguises in an attempt to recruit children.

**Fig. 4:**
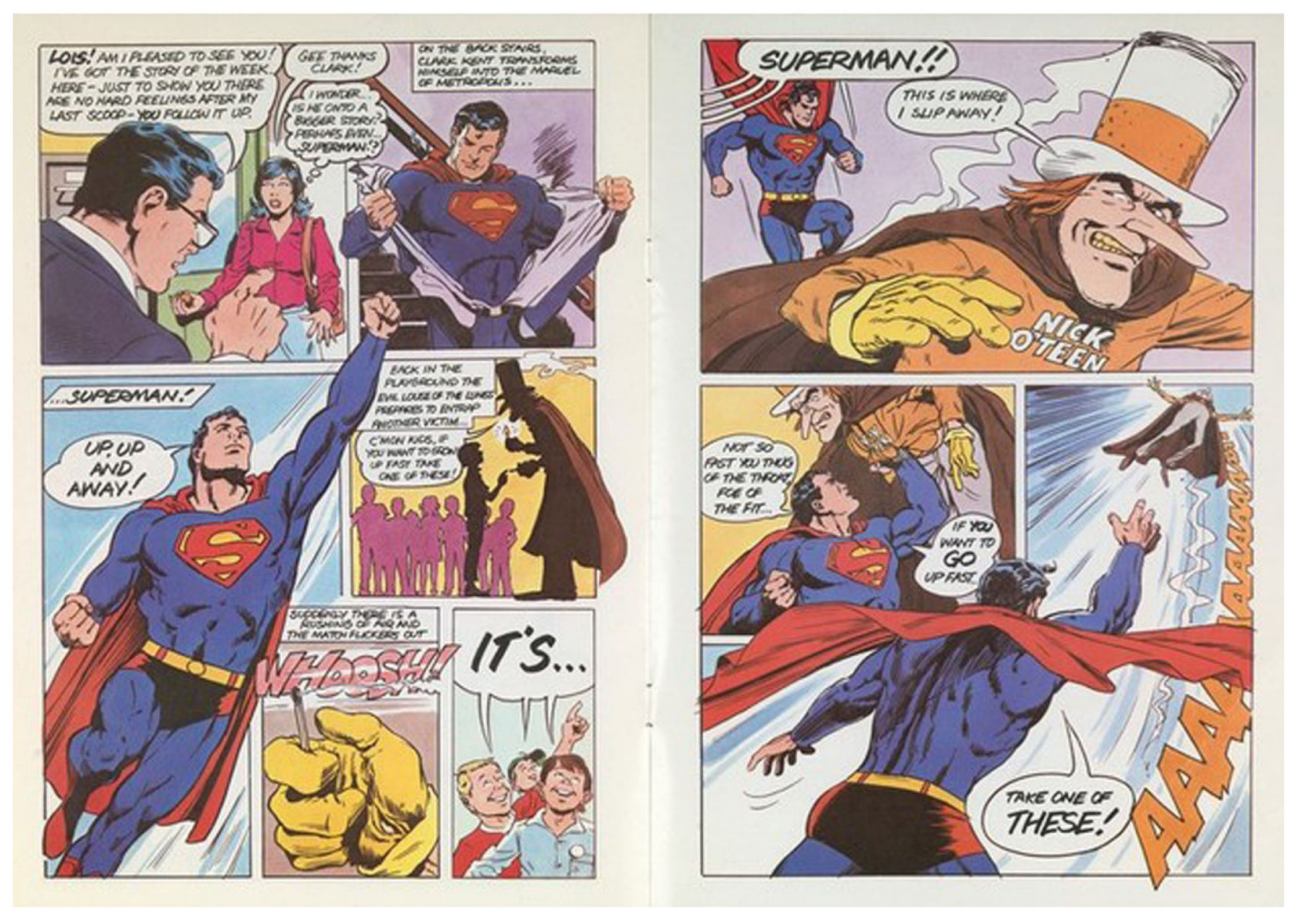
Pages 6 and 7 of the Superman vs. Nick O’Teen comic. Saatchi and Saatchi for the Health Education Council, 1980a, 1980b, 1980c. This figure is covered by the Creative Commons Attribution 4.0 International License. Reproduced with permission of Crown; copyright © Crown, all rights reserved. This information is licensed under the Open Government Licence v3.0. To view this licence, visit http://www.nationalarchives.gov.uk/doc/open-government-licence/. Image courtesy of the Wellcome Collection

The health message is also literally delivered by Superman himself using his traditional brand of trash-talk. When Superman grabs Nick O’Teen to prevent him leading children astray with more false claims that cigarettes ‘help you grow up fast’ Superman exclaims ‘not so fast you thug of the throat, enemy of the fit… if you want to GO up fast…’ then launches Nick O’Teen into the air (Fig. 4). As the HEC intended, Superman is being used here as a safe authority figure to deliver health messages without engaging the anti-authoritarian sentiment assumed to be an inevitable feature of children as a public. The use of a pun, albeit a clumsy one, helped to disguise the underlying health messages Superman delivers here with a little humour, while inviting children into an empathetic relationship with him as they laugh together at the vanquished Nick O’Teen.

**Fig. 5:**
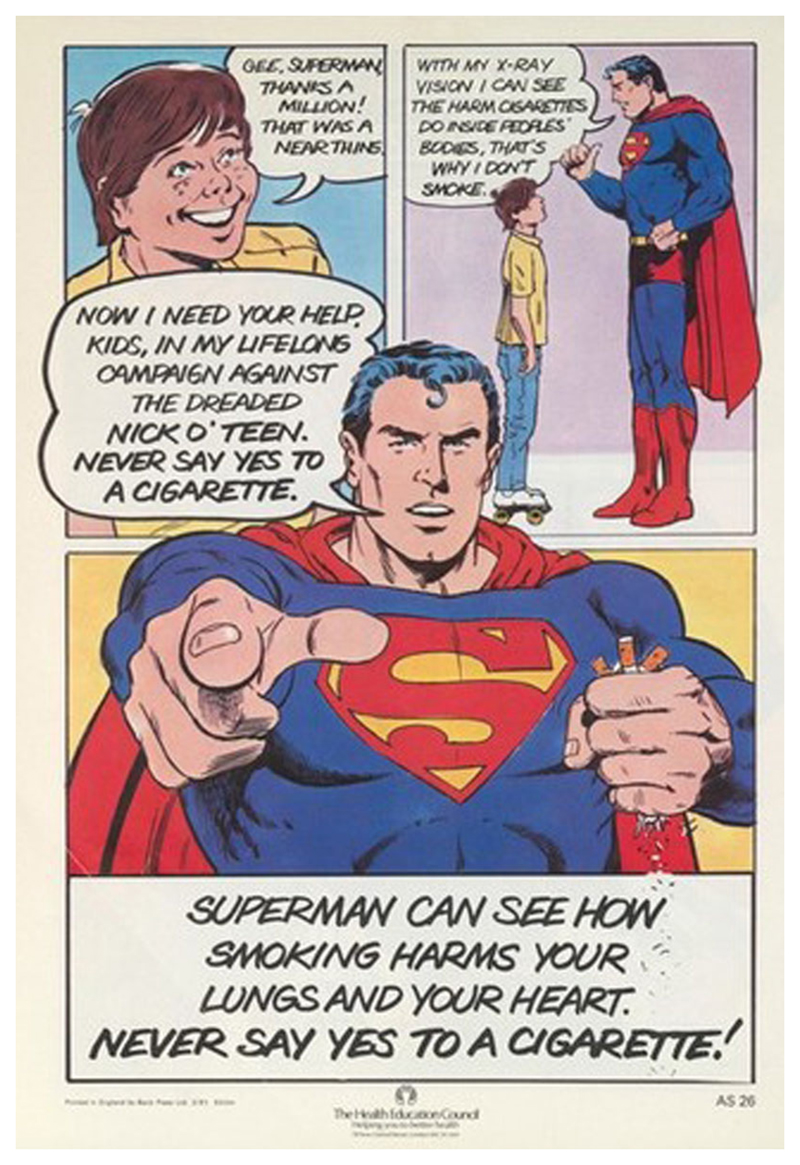
Final page of the Superman vs. Nick O’Teen comic. Saatchi and Saatchi for the Health Education Council, 1980a, 1980b, 1980c. This figure is covered by the Creative Commons Attribution 4.0 International License. Reproduced with permission of Crown; copyright © Crown, all rights reserved. This information is licensed under the Open Government Licence v3.0. To view this licence, visit http://www.nationalarchives.gov.uk/doc/open-government-licence/. Image courtesy of the Wellcome Collection

**Fig. 6:**
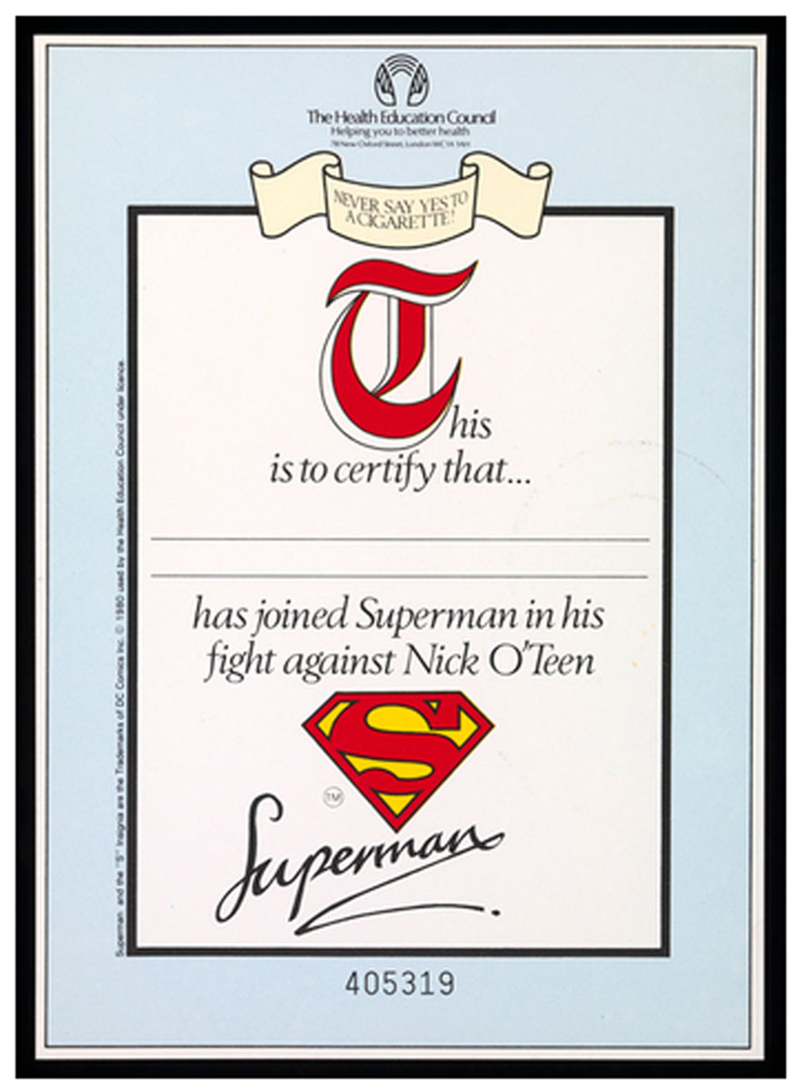
Certificate. Saatchi and Saatchi for the Health Education Council, 1980a, 1980b, 1980c. This figure is covered by the Creative Commons Attribution 4.0 International License. Reproduced with permission of Crown; copyright © Crown, all rights reserved. This information is licensed under the Open Government Licence v3.0. To view this licence, visit http://www.nationalarchives.gov.uk/doc/open-government-licence/. Image courtesy of the Wellcome Collection

Having saved the day, Superman offers up a straight-forward health message—‘I can see the harm cigarettes do inside peoples’ bodies, that’s why I don’t smoke’—which the children are depicted receiving gratefully (Fig. 5). This demonstrated to the intended audience how they should behave when furnished with the correct information. Not only are children told ‘Never say yes to a cigarette’ they are asked to join Superman’s ‘lifelong campaign’ and given examples of good behaviour to follow, in the heroic figure of Superman and the children who have denied Nick O’Teen. The HEC did not rely solely on rhetoric to try to persuade children that smoking was uncool or deleterious to health. As mentioned above, they offered them additional incentives through the Superman pack, poster competition and pledge certificate. While the poster competition engaged children in thinking creatively about how to dissuade smoking in others, recognising them as members of the public with something to offer, the certificate, in an echo of late nineteenth and early twentieth century temperance pledges (McAllister, 2012), offered ongoing membership in Superman’s ‘fight against Nick O’Teen’, asking children to look to their future and forswear smoking forever (Fig. 6). Of all the items in the Superman pack, the certificate provides the best evidence of the intention to create an ‘anti-smoking lobby’ of children. This points towards the fact that the campaign was not just about preventing individual children from taking up smoking, but about changing attitudes towards smoking more broadly. Indeed, children in this campaign had a multi-faceted role. At one level, the battle between Superman and Nick O’Teen was a classic representation of a fight between good and evil, with Superman saving children from the terrible fate of a lifetime of smoking. But the children represented in the images had agency, as did the children who interacted with the campaign both in its design, in its delivery, and its ongoing impact.

## Nick O’Teen: impact

The Nick O’Teen campaign certainly achieved a high degree of visibility. In the first two months, 200,000 children requested a pack, a number that rose to 800,000 by 1982. This equated to about one in ten of Britain’s child population. A series of surveys of random samples of children also suggested that the message got through to its recipients. A survey of 300 children who had filled in the coupon and received the pack found that 92% had retained the poster and 90% cited the message correctly or nearly correctly (Jacob, 1985). Another survey, conducted in 1983, almost a year after the last phase of the campaign ended, found that 73% of children were able to recall the main message without prompting (Carrick James Market Research, 1983). The HEC also took the fact that they had enquiries from 15 countries about the campaign, and similar efforts were mounted in Australia and Singapore, as evidence that this was a successful venture (Health Education Council, 1981a). There were some elements of the evaluation and response to the campaign, however, that might have given the HEC pause. When asked why they had sent off for the pack, 55% of children surveyed said that it was because they liked Superman, and 47% because they liked the anti-smoking message. When asked about the certificate, 48% saw it as enroling them in Superman’s fight against Nick O’Teen, 19% believed it was connected with them discouraging smoking in others, and just 15% saw it as a personal pledge never to smoke (Jacob, 1985). What this suggested was that some children may simply have sent off for the pack or put the poster on their wall because they liked Superman, and not because they were engaged with the campaign’s message. Superman himself was far from being a stable or neutral image strongly connected to anti-smoking. Indeed, there were other tropes associated with Superman that may have had the opposite effect. As at least two respondents to the HEC’s campaign pointed out, candy cigarettes bearing Superman’s image were on sale at the time of the campaign. The 1980 film Superman II featured a single cigarette brand on 22 separate occasions (Burki, 2017), and although Superman III (1983) could be thought of has having an anti-smoking message due to a plot device involving the replacement of an element of Kryptonite with tar from a pack of cigarettes, to some extent this was undermined by the clear presentation of the cigarette brand, an example of product placement by the tobacco company in question. No matter how carefully constructed, the HEC could not control the reception of its campaign or the meanings attributed to it by those who viewed it.

Assessing the long-term impact of the campaign, and especially the extent to which it was able to prevent children from taking up smoking, was also difficult. This was something that the HEC were well aware of. HEC Press Officer Micheál Jacob noted that ‘evaluation of a campaign like this is difficult, since its influence on behaviour can only be measured in the future and, even then, smoking behaviour is governed by a great many factors and subject to a great many influences.’ He went on to state that ‘It would be idle to pretend, and I think it is idle to pretend, that any health education mass media campaign on its own can make a significant long-term impact on health behaviour.’ Nonetheless, Jacob was confident that the overwhelming response by children, the favourable reception by schools, and the associated significant coverage by the mass media suggested ‘that the approach is one worthy of development’ (Health Education Council, 1981b). This somewhat down-beat view of the potential long-term impact of the campaign was to some extent borne out by the statistics on smoking. The percentage of girls aged 11–15 years that smoked increased between 1982 and 1986, from 11% to 12%, and the number of 15-year-old girls who were regular smokers from 25% to 27%. The percentage of boys who smoked declined, from 11% of boys aged 11–15 years in 1982, to 7% in 1986, with the number of 15-year-old boys who were regular smokers falling from 24% to 18% (ASH, 2018). Too much weight should not be put on such statistics, in part due to the small numbers involved, but smoking rates amongst children did not change much over the course of the next 30 years, suggesting that some groups of children were largely impervious to anti-smoking messages.

More broadly, the Nick O’Teen campaign took place at the same time as a wider crisis of confidence in the value of health education, and more specifically its ability to change behaviour. The Cohen committee and the establishment of the HEC in the late 1960s were indicative of optimism about health education, a wave that continued into the 1970s with the involvement of professional advertising agencies like Saatchi and Saatchi. By the 1980s, however, it was becoming increasingly clear that many of the issues that health education was supposed to reduce or prevent, such as heart disease, excessive drinking and obesity, were getting worse, not better. An editorial published in the *British Medical Journal* in 1982 argued that the HEC had achieved little since its establishment (Anon, 1982). Health educators themselves were developing a more critical view of their work which stressed the importance of social context and rejected a sole focus on individual behaviour change (Rodmell and Watt, 1986). Other approaches to understanding how the public responded to health education messages were beginning to be developed, ones that attempted to get to grips with lay understandings of these, rather than assuming that the message was received unaltered. In a piece of research conducted shortly after the Nick O’Teen campaign concluded, a multi-disciplinary team including an anthropologist and an epidemiologist looked at the response to a health education initiative conducted in South Wales around heart disease. The researchers found that the public interpreted the messages, and specifically the presentation of the risk of having a heart attack, within their own framework. This was related to the experience of friends and family, as well as images on TV and in magazines (Davison, 1989; Davison et al., 1991; Frankel et al., 1991). The public’s understanding of public and individual health was complex, multifaceted and rooted in a wider cultural and social context (Mold et al., 2019). Indeed, narrow debates about ‘success’ or ‘failure’ in terms of reducing smoking rates or preventing lung cancer miss the real message of health education: the ways in which it constructed its publics and they constructed it.

## Children: a problematic public

The design, delivery and reception of the Nick O’Teen campaign reveals much about the especially problematic nature of children as publics. Child health had long been an obsession of public health officials. Throughout the nineteenth and early twentieth century considerable effort was directed towards improving infant and maternal health (Apple, 1995; Davies, 1988; Welshman, 1997). Much of this was framed around eugenic concerns about the future health of the ‘race’. There were often sharp gender divisions, with boys’ health seen through the lens of fitness and masculinity, and girls’ health around future motherhood, although this did start to change in line with broader shifts in the position of women in society (Heggie, 2008; Marland, 2013). Notions of the future continued to be crucial to perceptions of child health, but also to children as future citizens in the post-war period (King, 2016).

As already hinted at, ideas about the future figured centrally within the Nick O’Teen campaign. The entire initiative was aimed at prevention—a forward looking view—but a particular notion of children, the family, and the future was at work here. The ambivalent and multifarious attitudes to children as a public demonstrated by the HEC in their construction, delivery, dissemination and assessment of the Nick O’Teen campaign was indicative of the constructed and contested nature of childhood in a general sense, but also more specifically those of the 1980s (Aries, 1996, pp. 9–11; Thomson, 2013, pp. 153–183). Children were deployed in the discourses of the New Right as both an emotive talisman and cornerstone of the nuclear family (Elizabeth, 2016, 2012). For the Conservative party under Margaret Thatcher, an idealised nuclear family, with children at its heart, provided both a structuring metaphor and target for New Right policies, argued for with reference to the recurring and adaptable mantra: ‘What is right for the family is right for Britain’ (Thomas, 1993). Using the ‘privatised family’ as a bracketing device, the Conservative party were able to break with the post-war consensus and intimate that Welfareism was intrusive and infantilising, and thus an institution at odds with an imagined idealised ‘stable, self-reliant, moral, nuclear family’ (Nunn, 2001, pp. 16–17). The citizen’s relationship with the Welfare State was recast as ‘coddling’ rather than care, and the consumer society, with its emphasis on independence and responsibility, was proposed as its moral replacement (Hall, 1983, p. 29). This placed public health interventions, especially those seeking to intervene in the lives of children, and by extension the private realm of the nuclear family, on shaky ground. Instead of protecting the future through preventative policies, state interventions threatened it by incurring dependence and destabilising the nuclear family through interference.

Touring the United States as the new leader of the Conservative Party in 1975, Margaret Thatcher promised a future where the New Right would free children from the malevolent influences of a society controlled by those seeking ‘my rights at all costs regardless of who has to pay’ (Thatcher, 1989), returning children to their ‘rightful owners’, outside state interference (Nunn, 2001; Winter and Connolly, 1996, p. 29). The child in this conception performed a dual representative function; firstly as an idealised powerless innocent, desirable to parents as a site onto which all moral and monetary aspirations may be bestowed and so the future guarded and shaped; and secondly as the embodiment of childhood, an undesirable intermediate time of vulnerability where all agency belongs to one’s guardians. As a framing device, the figure of the child thus embodied many of the conflicts which lay at the heart of the ‘unstable amalgam’ between neo-liberal economics and authoritarian moral conservativism which marked the Thatcher and Major governments (Kingdom, 1992, p. 2). Indeed, as a consequence of this rhetoric, the needs of the child were offered up as the motive behind a number of conservative legislative moves which defined the limits of the state, the private sphere, the individual and the public good. However, despite the dominance of the morally conservative right, the 1970s and 1980s also saw inroads made by the children’s rights movement. Campaigns focused on child labour, corporal punishment and children’s rights in hospital and state care, successfully placed the needs of the child with agency (as well as the passive vulnerable child), firmly on the agenda (Franklin and Franklin, 1996, pp. 96–97). A consequence of these fraught debates (and the legislative changes which were in some cases their consequence) was a framing and reframing of the figure of the child, and a reimagining of the limits of what was expected of it and for it. Indeed, late twentieth century legislation constructed children within an educational context as ‘ideally non-sexual, vulnerable and dependant’, their agency and access to information ‘restricted or censored’ by the desires of parents, while in a medical context they were constructed ‘as “quasi-adults” entitled to confidential advice and treatment’ able to refuse or accept medical interventions through informed consent (Blair and Monk, 2009). While vulnerable conceptions of the child certainly persisted and were reinforced in this period, the 1970s and 1980s also saw the child imagined anew as a discerning consumer and possessor of rights.

This multiplication of possibilities is evident in the Nick O’Teen vs. Superman campaign. The vulnerable child certainly appears in the Nick O’Teen narrative, but a child with agency, and so the moral responsibility to join Superman’s fight against smoking, also emerges in the text. Furthermore, a child who consumes comics and can access a certain authority within its family, and is able to persuade parents and others not to smoke, is also discernible in the HEC campaign files. A conflicting view of the child was therefore in operation. This child was simultaneously presented as vulnerable to persuasion and in need of protection, but also potentially an anti-smoking lobbyist; as a bad future consumer of cigarettes and potential threat to public health, and as a good obedient future citizen who refuses to smoke. Running alongside and interacting with the Nick O’Teen’s campaign’s construction of the future was another narrative about children as a group to be protected from external threats and as citizens with agency. On one level, the Nick O’Teen materials told a story about children being tricked by an evil force into smoking, a danger that they were rescued from by the heroic figure of Superman. Without his intervention, it is implied, the children would have started smoking. But there were clear notions of agency at work here too. The implementation and delivery of the campaign involved children in the ‘fight’ against Nick O’Teen. Children were not just passive recipients, but active participants in the campaign. Moreover, children were seen as being able to recruit other children in this fight, and even work towards influencing their parents’ smoking behaviour. The view of children as expressed in the Nick O’Teen campaign was thus paradoxical: they were simultaneously helpless dupes and active agents.

## Conclusion

The paradoxes and conflicts that ran through the Nick O’Teen vs. Superman campaign were not unique to this campaign or its imagined public. After nearly twenty years of effort, doubts about the ability of health education to change individual behaviour were beginning to set in. These concerns revolved not only around the effectiveness of the materials, but about the agency of the publics who interacted with them. The public could ignore, resist or re-appropriate health education messages. In many ways, the same was true of campaigns like Nick O’Teen vs. Superman that focused on children and young people, only more so. Fears about the potential vulnerability of children on the one hand, and their unruly nature on the other, were an exaggerated version of concerns about the public as a whole. The child-public anticipated by the Nick O’Teen campaign was also reflective of the demographic targeted and influences of the time at which it was being constructed. Unlike child health education campaigns of the past, the Nick O’Teen campaign ascribed children a greater degree of capacity to make their own decisions and influence others. Yet, the agency of this child-public was constrained, limited by longer-running fears about the vulnerability of children and their futures. Broader concerns about the nature of childhood in the 1980s were also on display, especially around the breakdown of the nuclear family and the growing sense of children as individuals with rights and the ability to exercise these. These somewhat contradictory concerns about the danger of youthful agency and the vulnerability of childhood can also be found in later 1980s and 1990s health campaigns targeted at children and adolescents. For example, in AIDS-related education materials we see children forewarned against the dangers of unprotected sex (an external threat), without being provided with any real explanation of the safer-sex practices which might protect them for fear that such knowledge might engender undesirable sexual behaviours (Elizabeth, 2016).

The Nick O’Teen vs. Superman campaign took place at a critical juncture not only for conceptualisations of children, but of the wider public and its health too. As the growing disillusionment with health education focused on individual behaviour change made clear, the idea that a single-unitary public could be made healthier through such campaigns was beginning to breakdown. Although public health practitioners had long been aware that there was not one public but many, campaigns like Nick O’Teen vs. Superman were increasingly being designed in such a way that allowed for the agency of their recipients. In this, then, we can see signs of moves towards a reimaging of the public as a set of what Hinchcliffe and colleagues call ‘healthy publics’. Although views of the target audience of the Nick O’Teen campaign were still some way from the notion of ‘healthy publics’, the HEC’s desire to work with children in designing the campaign and to involve them in its delivery point to the development of a more sophisticated understanding of ‘the public’ or particular publics. Yet, this complex conceptualisation of multiple publics interacted with another set of tendencies that served to flatten ‘the public’ and its interests. Claims made in the name of defending or improving ‘public health’ continued to skirt over the diversity of publics and needs involved. Superman may have defeated Nick O’Teen, but the battle for who defines the health of the public, and how this should be achieved, was far from over.
